# A patient-oriented clinical decision support system for CRC risk assessment and preventative care

**DOI:** 10.1186/s12911-018-0691-x

**Published:** 2018-12-07

**Authors:** Jiannan Liu, Chenyang Li, Jing Xu, Huanmei Wu

**Affiliations:** Department of BioHealth Informatics, School of Informatics and Computing, Indiana University Purdue University Indianapolis (IUPUI), Indianapolis, IN USA

**Keywords:** Colorectal Cancer, CDSS, Risk factors, Visualization

## Abstract

**Background:**

Colorectal Cancer (CRC) is the third leading cause of cancer death among men and women in the United States. Research has shown that the risk of CRC associates with genetic and lifestyle factors. It is possible to prevent or minimize certain CRC risks by adopting a healthy lifestyle. Existing Clinical Decision Support Systems (CDSS) mainly targeted physicians as the CDSS users. As a result, the availability of patient-oriented CDSS is limited. Our project is to develop patient-oriented CDSS for active CRC management.

**Methods:**

We implemented an online patient-oriented CRC CDSS for the public to learn about CRC, assess CRC risk levels, understand personalized CRC risk factors, and seek professional advices for people with CRC concerns. The system is implemented based on the Django Model-View-Controller (MVC) framework with an extensible background MySQL database. A CRC absolute risk prediction model is applied to calculate the personalized CRC risk score with a user-friendly web survey. An interactive dashboard using advanced data visualization technics will display and interpret the risk scores and factors. Based on the risk assessment, a structured decision tree algorithm will provide the recommendations on customized CRC screening methods. The CDSS also provides a search function for preferred providers and hospitals based on geographical information and patient preferences.

**Results:**

A prototype of the patient-oriented CRC CDSS has been developed. It provides an open assessment of potential CRC risks via an online survey. The CRC risk predictive model has been implemented. The prediction outcomes of risk levels and factors are presented to the users through a personalized interactive visualization interface, to guide the public on how to reduce the CRC risks by changing their living styles (such as smoking and drinking) and diet characteristics (such as consumptions of red meat and milk). The CDSS will also provide customized recommendations on screening methods based on the corresponding risk factors. For users seeking professional clinicians, the CDSS also provides a convenient tool for searching nearby hospitals and available doctors based on the location preferences and providers characteristics (such as gender, language, and specialty).

**Conclusions:**

This CRC CDSS prototype provides a patient-friendly interface for CRC risk assessment and gives a personalized interpretation on important CRC risk factors. It is a useful tool to educate the public on CRC, to provide guidance on minimizing CRC risks, and to promote early CRC screening that reduces the CRC occurrences.

**Electronic supplementary material:**

The online version of this article (10.1186/s12911-018-0691-x) contains supplementary material, which is available to authorized users.

## Background

Colorectal Cancer (CRC) affects caecum, colon, and rectum, which is the third leading cause of cancer death among men and women in the United States [1]. The lifetime risk of developing CRC is about 1 in 21 (4.6%) for men and 1 in 24 (4.2%) for women. It is estimated to have 135,430 new diagnosed cases and result in 50,260 deaths in 2017, accounting for the 9% of cancer deaths. The mortality rates have been decreasing for several decades because of changes in risk factors such as the introduction and dissemination of screening tests, and improvements in treatments [2–4]. Statistics showed that between 66 and 75% of CRC cases could be avoided with a healthy lifestyle [5] and appropriate dietary changes. Regular physical activities and maintenance of healthy weight could substantially reduce the morbidity and mortality associated with colorectal cancer [6]. There are many researchers worked on CRC risk factors and CRC risk scores calculations [7–9]. One is the absolute risk score calculation model by Andrew et al.’s [8] to be discussed further, which is adopted in our work.

However, the public knowledge on the significance of CRC is limited. Many do not recognize the significant impact of lifestyle on the development of CRC. It is the essential motivation for this project on constructing the patient-oriented CDSS. Currently, CDSS is serving an important role in patient visits, it was reported that 30% of annual US patient visits will use Electric Health Report (EHR) systems and 57% of EHR involved patient visits will use CDSS [10]. Several CDSS features such as automated decision support as part of workflow, provision of recommendations, have been proved to improve patient care significantly [11]. Previous study has also shown that 92% of existing CDSS enrolled physicians as primary users [12], the number of patient-oriented CDSS is very limited and there is no research on patient-oriented CDSS specialized in CRC. Therefore, a CDSS for CRC risk assessment, education, and preventative care, that are not only open to the public access but also connected to EHR system will play a critical role in the preventative care of CRC patients.

## Methods

### The underlying algorithms of the CDSS

The group first conducted literature reviews of the potential CRC risk factors, CRC risk score calculation, and CRC screening approaches. We adopt the absolute risk score calculation model [8] for the CRC risk score calculation in our CDSS. It used population-based case-control studies as source data to train a prediction model for estimating the risk of developing CRC in a certain period (e.g., 10 or 20 years). In this model, the projected probability will be the absolute risk score with a confidence interval of 95%. Eq. 1 summarizes the primary components of their model:

$$ absolute\ risk={f}_1\left( relative\ risk\ parameters\right)+{f}_2\left( age\  specific\ cancer\ hazards\right)+{f}_3\left( attributable\ risk s\right) $$

The detailed mathematic model and risk factor coefficients have been explained in Freedman’s report [8]. The relative risk parameters are estimated from population-based case-control data. Sample risk factors include the numbers of relatives with CRC, the patient physical activity, smoking habit, diet preference, body mass index, and others. The f_1_() function will calculate the relative risk based on tumor sites, including the proximal (cecum through transverse colon), distal (splenic flexure, descending, and sigmoid colon), and rectal (rectosigmoid junction and rectum) tumor sites. The f_2_() is a function to predict the CRC risk based on different ages and risk factor profiles. The f_3_() function will assess the attributable risks from the case-control data., The baseline age-specific cancer hazards and attributable risks are all estimated from the case-control data. The final CRC absolute risk predicted by this model combines the three absolute risks (proximal, distal, rectal) and risks of competing causes of death other than CRC. A SAS Macro program which implemented the proposed model is publicly available online. This program eased our effort on integrating the absolute risk score calculation model into our CDSS.

In our CDSS implementation, we adopted the 20-year absolute risk score as the projected risk score. We then rescaled the absolute risk reported by Andrew’s model to a range of [0, 10] based on the maximum and minimum risk scores. Based on the risk scores, the CRC risks are classified into three levels, according to a previous study [9] by Jane et al. The low-risk level, medium risk level, and high-risk level, reported from our CDSS system, are corresponding to the scaled risk score ranges of [0, 3], (3, 7], (7, 10], respectively. For example, if the rescaled risk score is higher than 7, our CDSS will report high-risk score.

Our developed CDSS also provides the recommended CRC screening methods. Information on multiple screening methods that are suitable to the identified CRC risk factors are gathered. The screening method details, such as the performance complexity and test time intervals, are stored in the backend database and used for giving screening recommendations to patients based on their risk factors. The recommendation algorithm is a simple structured decision tree [13]. For instance, if a patient reports that he/she has inflammatory bowel disease, the decision tree will report Fecal Immunochemical Test (FIT) as one of the recommended screening methods because of its low complexity, low side effect, and low cost.

### The application framework

**Fig. 1:**
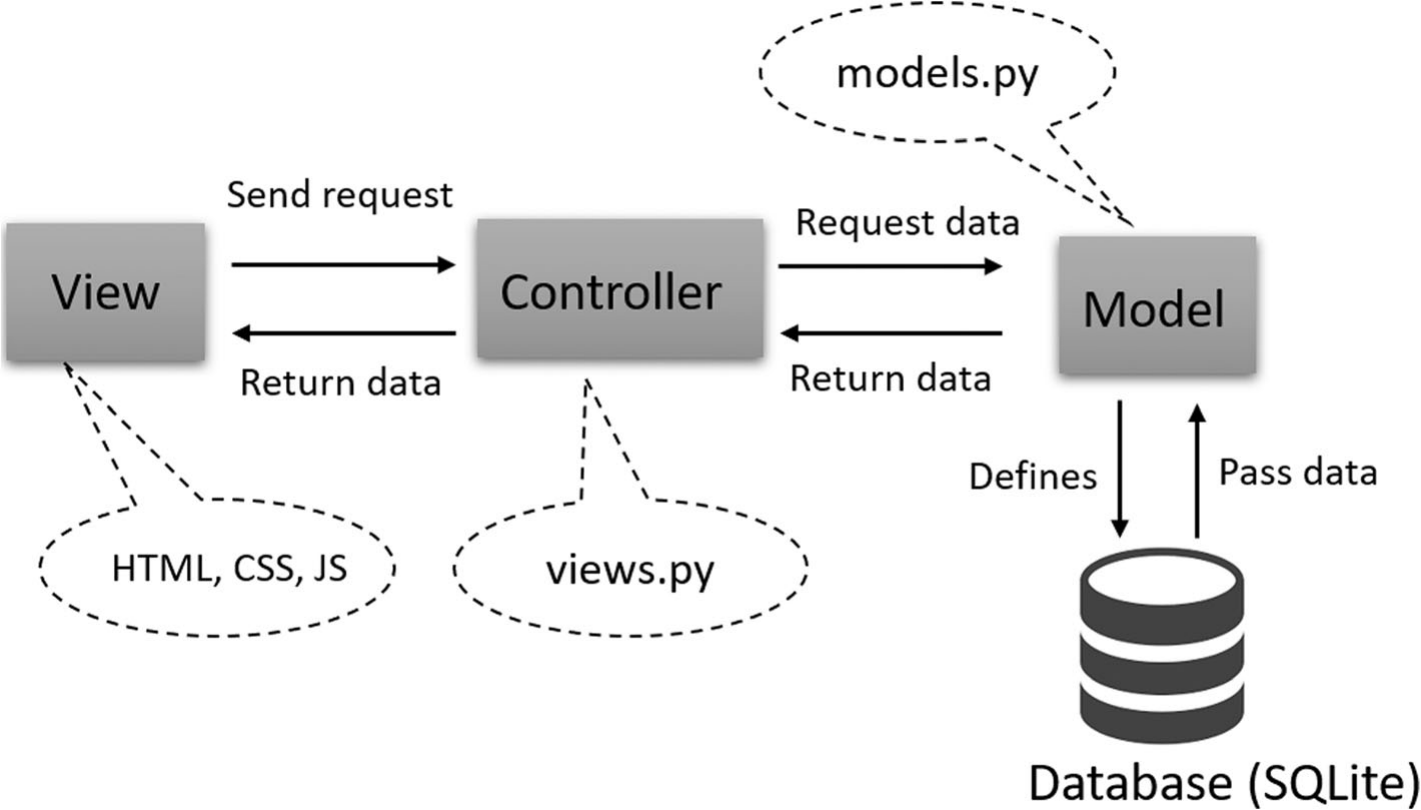
The Django MVC framework

Figure 1 illustrates the system infrastructure for the prototype of the patient-oriented CDSS. It is developed using Django, a Python-based Model-View-Controller (MVC) web application development framework. An MVC framework separates application functionalities into three domains. The *models* describe the data structures of the backend database. The *views* display application outputs and collect inputs, which can consist of several files, such as HTML, CSS, JavaScript, and others. The *controllers* define the internal logic of the application. It is also responsible for data processing [14]. In our CDSS, we use the D3 data visualization package to visualize the risk score data as bullet chart and create the interactive dashboard [15].

### The backend database

**Fig. 2:**
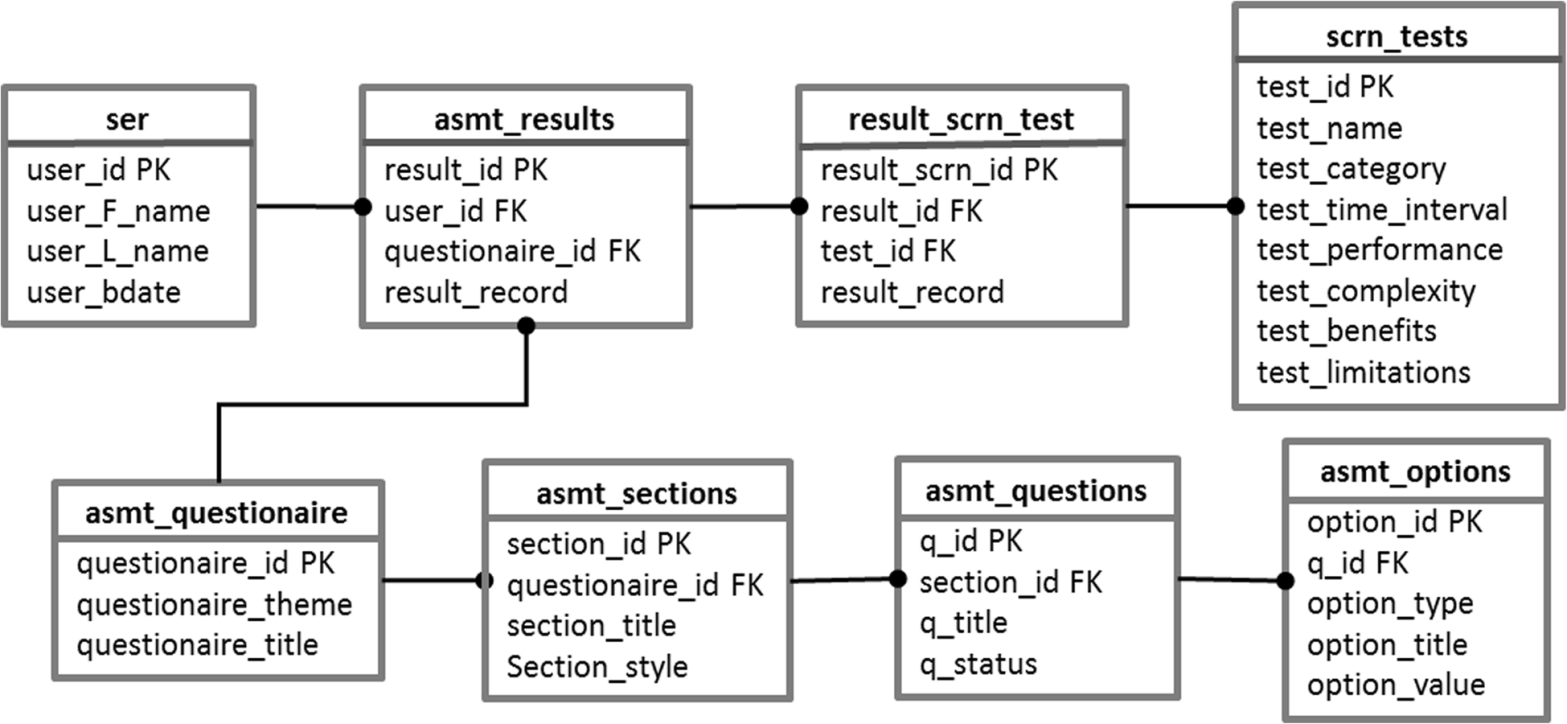
The entity relationship diagram for the backend database

We use MySQL [16] as the backend database. Figure 2 shows the primary data structure. The *User* table stores the information of the CDSS users. When the CDSS is connected to an EHR system, the user information can be transformed to a patient table in the EHR system. The *asmt_results* table is the main component that stores the assessments patients have done. Since our CDSS will give recommendations on CRC screening methods, the *result_scrn_test* table serves as a relation table, which represents a many-to-many relationship between assessment results and screening methods. All the detailed information (e.g., test name, test time interval, test performance, etc.) on screening methods will be kept in the *scrn_tests* table. The *asmt_questionnaire* table defines questionnaire title and theme. In each questionnaire, there will be several sections, which contain some similar type of questions. The *asmt_sections* table describes the section title and its preferred style. All the questions will be stored in the *asmt_questions* table. Each question has a status attribute, which has a potential value of active or disabled. This attribute will help CDSS administrator add or delete questions for each questionnaire easily, making the database design more flexible and extensible. Options for each question will be kept in the *asmt_options* table, with a type attribute to indicate the type of input (such as a radio button or a text input) and a value indicating the risk score for each risk factor. The full list of attribute descriptions can be found in Additional file 1.

The data structure of our database keeps all the relative information used by our CDSS. It also keeps the flexibility of changing questions and options in the questionnaire. By designing such a database structure, we have also maintained the flexibility for CDSS upgrades in the future.

### The website design

**Fig. 3:**
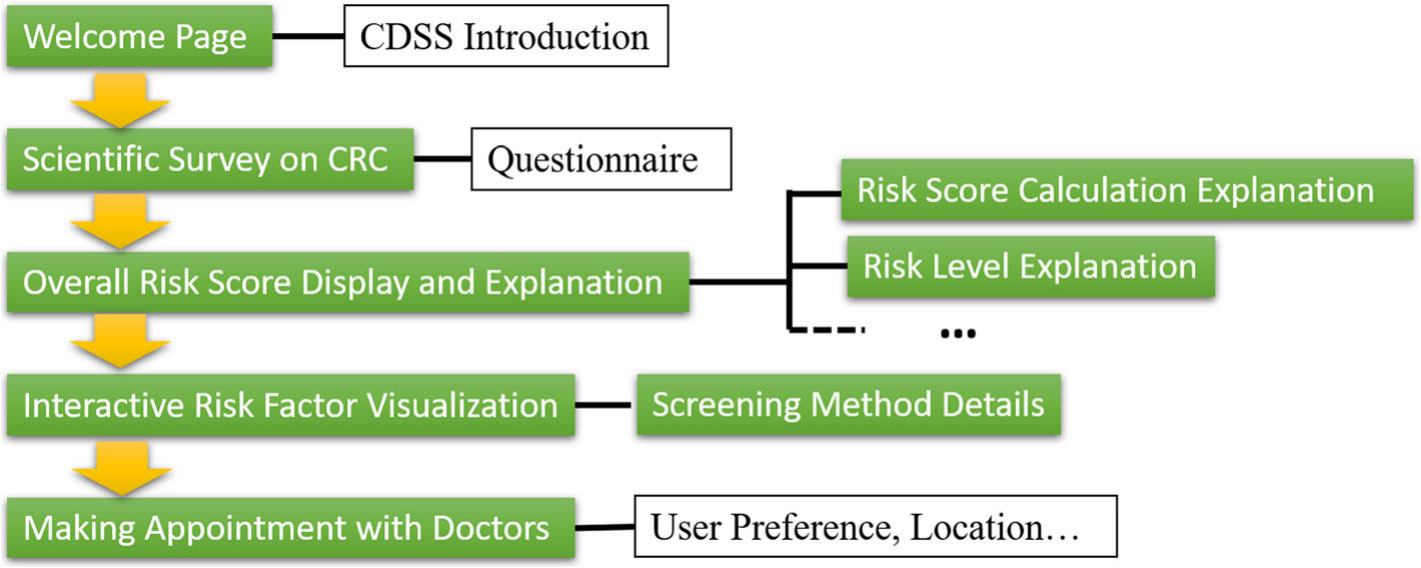
The design and workflow of the CRC CDSS website, where the green boxes indicate a functional webpage and the white boxes describe the content of the relative webpage. The arrows show the workflow of the whole website

Figure 3 demonstrates the design and workflow of the CRC CDSS website. The green-colored textboxes indicate a webpage in the CDSS. Other boxes describe the content of the pages. Our CDSS prototype has the following components and primary CDSS functions. The first is an interactive website with an anonymous scientific questionnaire to obtain the information about essential CRC risk factors. These questions are designed according to previous studies on CRC risk factors [8]. The second is a user-friendly display module with the risk scores calculated based on the input risk information. The third innovative component is an interactive visualization dashboard to show how changing lifestyle habits and diet preferences will affect their CRC risk level. The visualization is personalized based on the user input to the survey questions. Fourth, we incorporate a CDSS module to provide individualized recommendations on screening methods based on survey results and risk scores. The fifth is an appointment scheduling system with CRC providers based on user preferences on doctor characteristics and geographical locations. Last, the CDSS provide educational information on CRC preventative care.

## Results

**Fig. 4:**
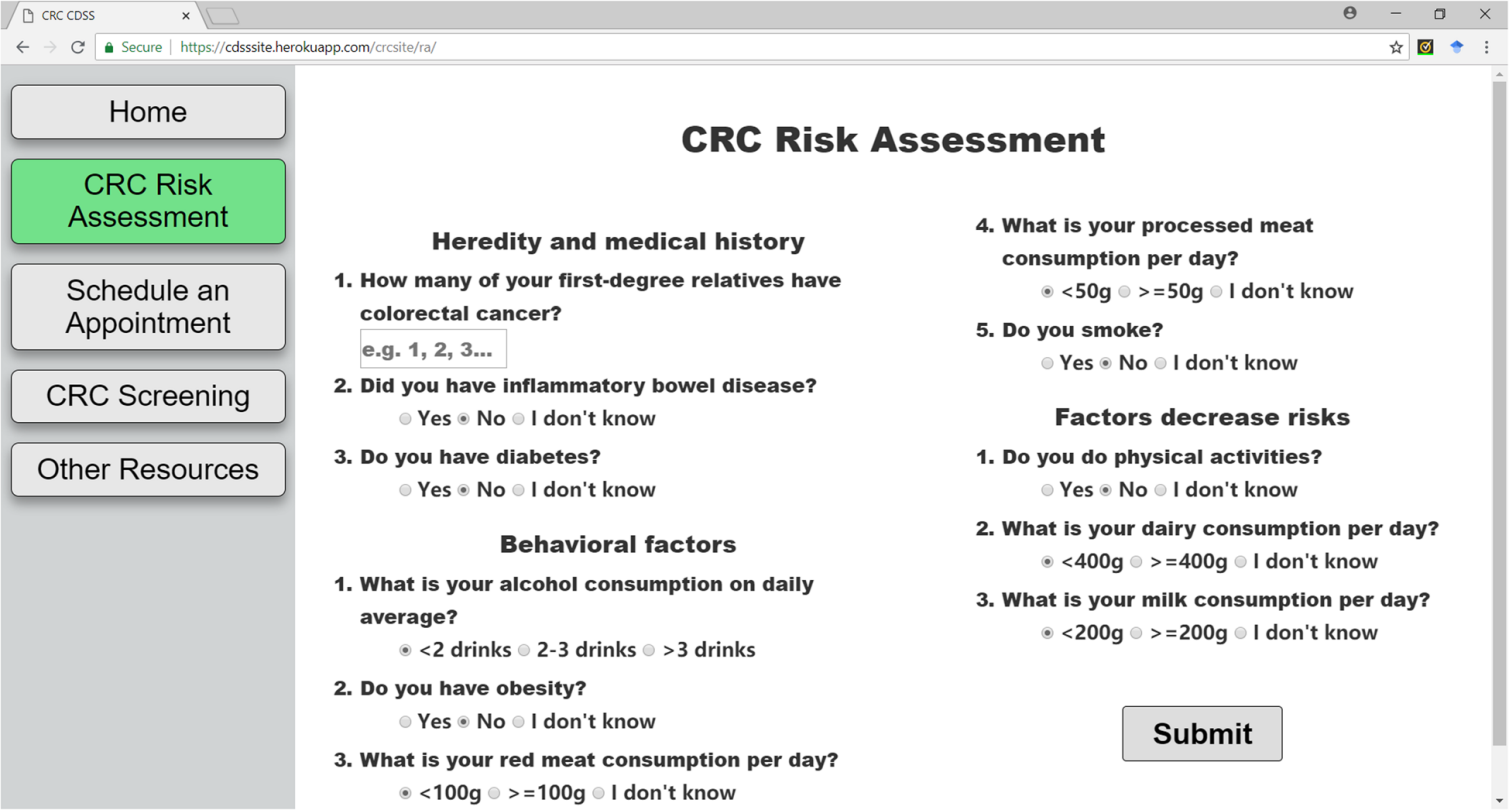
The interface for survey CRC risk factors. Left side of the webpage is navigation bar of whole site. All questions are categorized into different sections

**Fig. 5:**
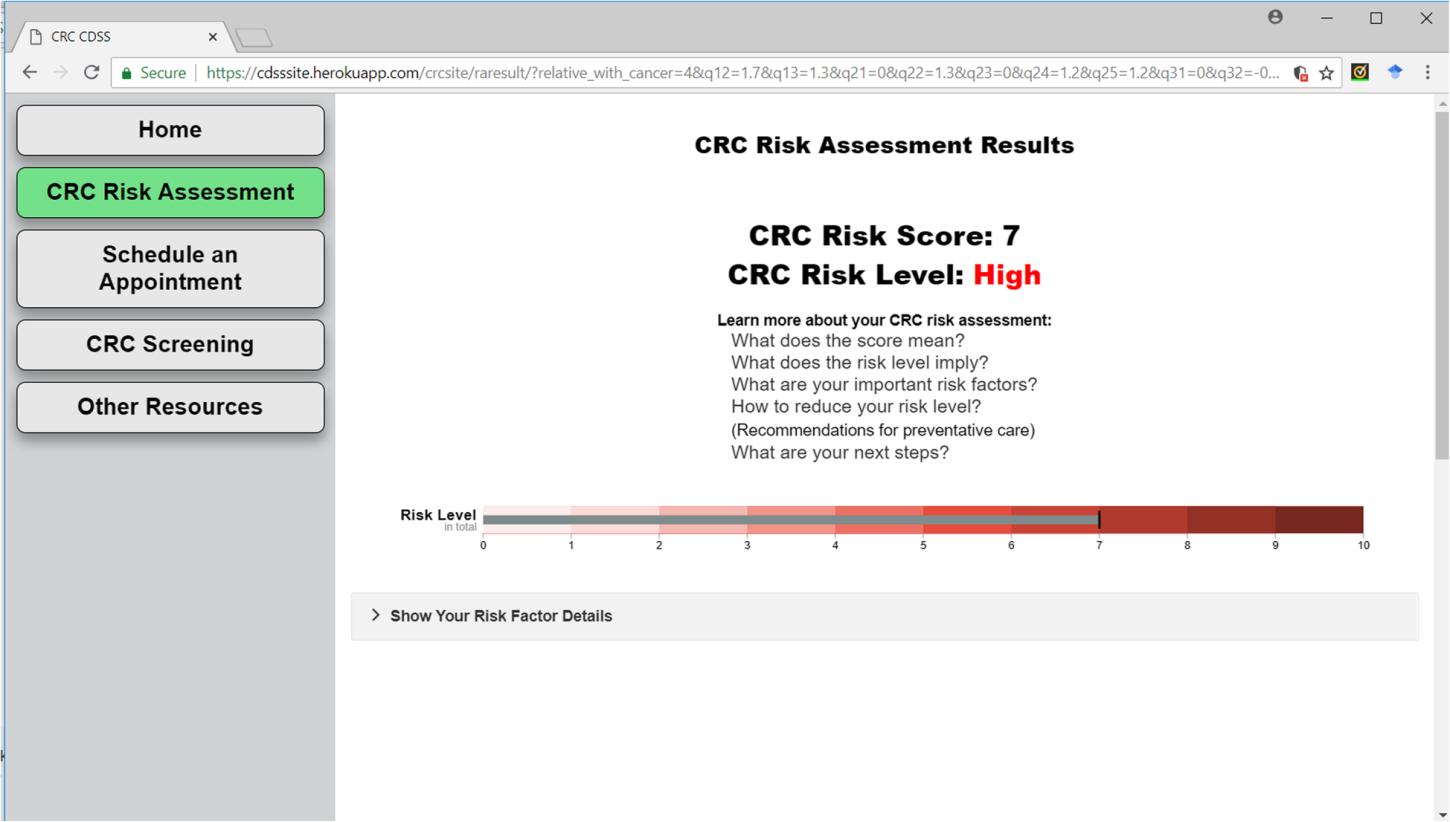
CRC risk assessment results. The top part shows the risk score and risk level based on the user survey input. A bullet chart is used to visualize the risk score in a range of 0 to 10. Several useful links are provided above the bullet chart

We have designed an interactive website to provide an easy-to-use questionnaire for potential risk factors of the users, as illustrated in Fig. 4. After completing the survey, the CDSS will first report the overall risk score with a bullet chart to visually display the users their risk levels, as shown in Fig. 5. The CRC risk level has different highlighted colors according to different risk levels. Several useful links are provided to help the users to understand the risk scores and risk levels.

**Fig. 6:**
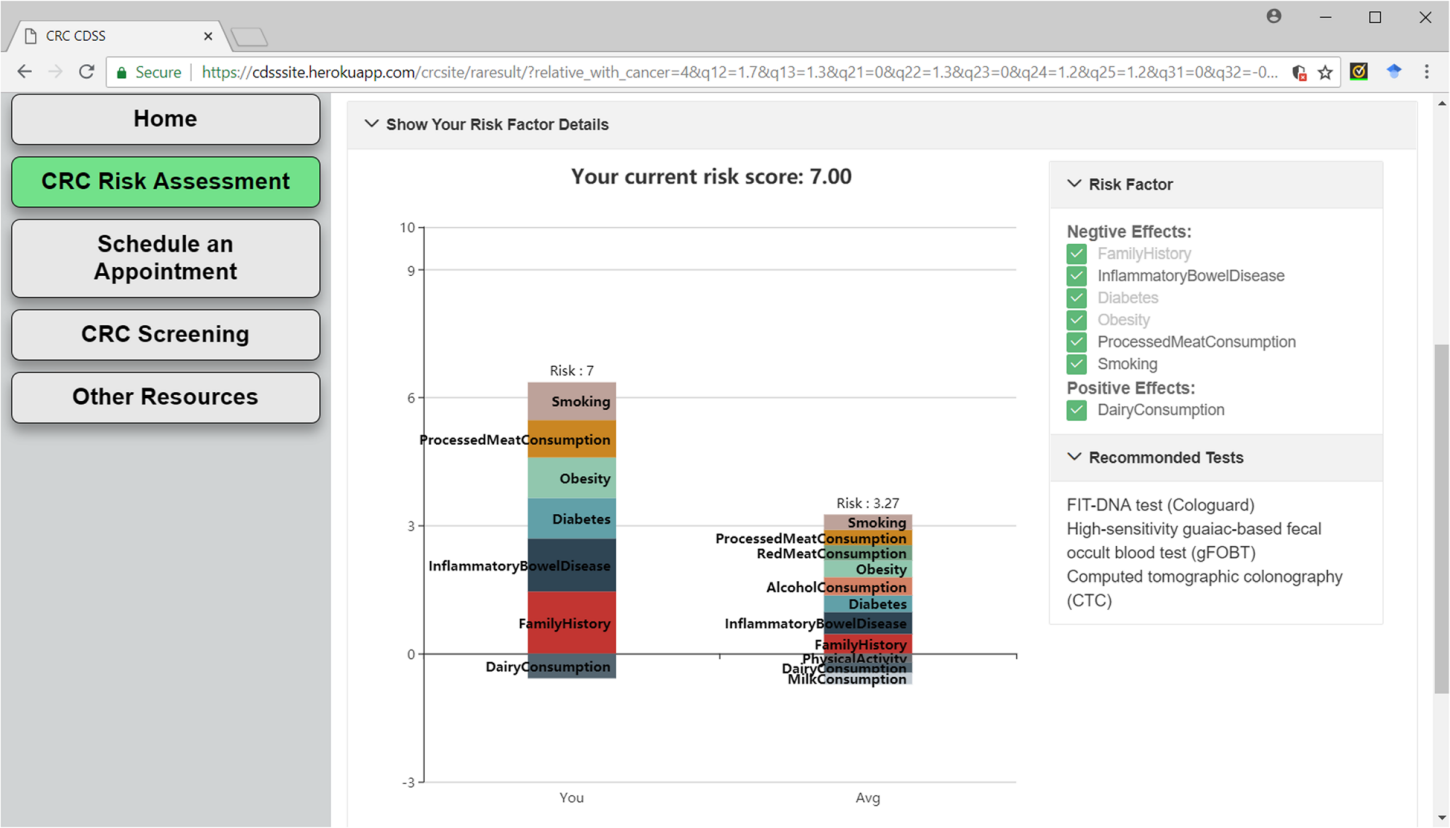
The interactive visualization of risk factors. In the stacked bar chart panel. The left bar of the stacked bar chart shows the proportion of each CRC risk factor of the user against the highest risk score (at 10). The right bar gives an average value of each CRC risk factor according to populations in the database. The right upper panel provides a list of user reported risk factors. The user can uncheck/re-check the modifiable risk factors to observe the change of total CRC risk score. The risk score will change simultaneously when the user unchecks/re-checks the reported risk factors

The salient feature is the interactive stacked bar chart, generated according to user inputs. The stacked bar chart shows the total score on the top of each bar, with different risk factors. A user would be able to understand the impact of each risk factor interactively and visually. As illustrated in Fig. 6, the interactive visualization interface will allow a user to modify their preferences on the side buttons to observe the dynamic changes of risk scores. It helps them to decide what healthy lifestyle (such as smoking vs. non-smoking, drinking milk or not) will reduce their CRC risks. On the other hand, risk factors such as family history cannot be modified since a user cannot change this kind of risk factors. Thus, these factors are not clickable.

On the side of the stacked bar chart, we provide recommendations for CRC screening methods, which are ranked using the simple decision tree method based on the questionnaire input and total risk score. Every recommendation method is a clickable link, which will lead users to an information page with detailed description on recommended screening method.

**Fig. 7:**
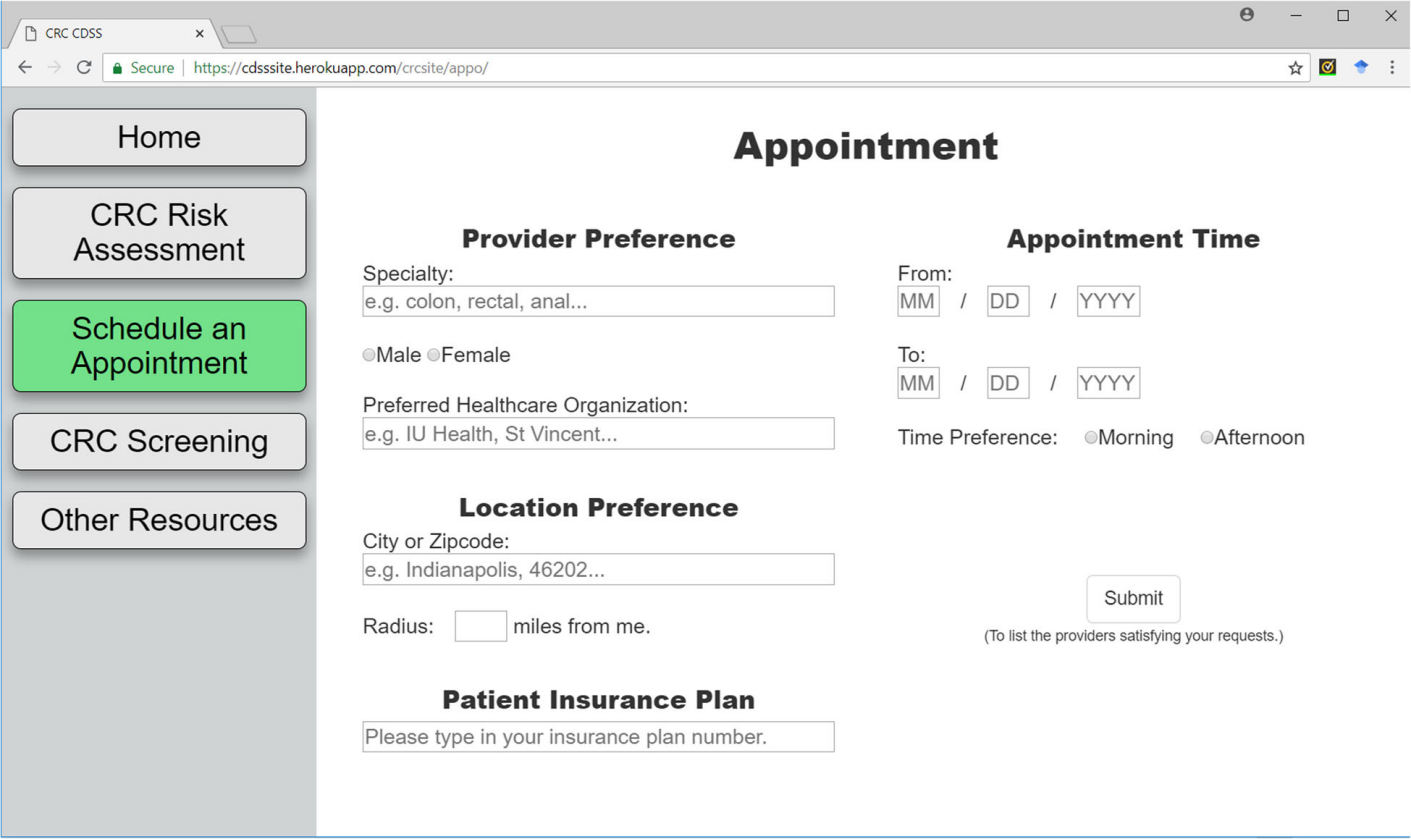
The dashboard for making an appointment. Basic preferences on hospitals and provider information should be provided in this page to obtain a list of available doctors

**Fig. 8:**
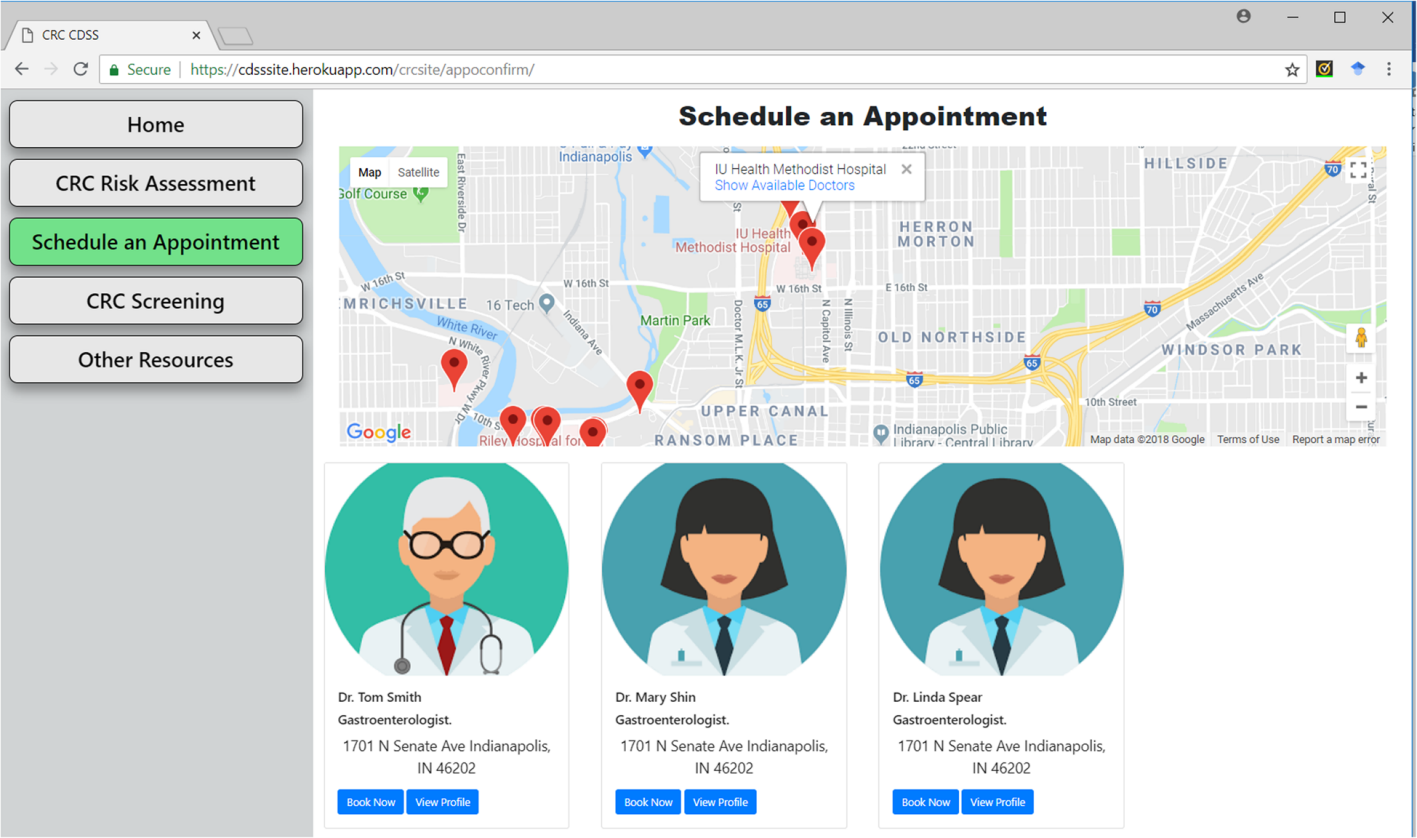
The results of the nearby hospital search and doctor availability. After providing user preferences, our CDSS will search hospitals by zip code and find available doctors matched the preference in each hospital

To schedule an appointment with healthcare provider, the CDSS provides options for user inputs, such as location and doctor preferences, as manifested in Fig. 7. Based on the information, the CDSS offers recommendations on which hospitals or community health centers a user can visit. It also lists the providers available for appointment. This page is designed to connect to hospitals and community health centers’ scheduling systems to get information about available doctors. For the testing purposes, we populated the system with sample hospitals in the Indianapolis area and synthesized doctors to simulate and test the system function. The Google map API is applied to achieve the hospital search function based on zip code, as illustrated in Fig. 8.

## Discussions

Currently, there are several available online tools for CRC risk score prediction. Colorectal Cancer Risk Assessment Tool (CCRAT) (https://ccrisktool.cancer.gov), sponsored by the National Cancer Institute (NCI), provides an interactive tool to help estimate a person’s risk of developing CRC. It has a well-designed questionnaire to collect related information. The CRC risk calculation also follows the Freedman algorithm [8]. However, the CCRAT only displays the risk calculation results in an absolute percentage, which is hard for users to understand. Second, the simple bar chart for result presentation is the overall risk, lacking the detailed information on various individual risk factors and their impacts on the overall CRC risk. Another CRC risk calculation tool is the Colorectal Cancer Predicted Risk Online (CRC-PRO) [17], which can be used to calculate 10-year CRC risk score. It has an easy-to-use interface. However, the CRC-PRO only presents the calculated probability without any interpretation of the result. It is difficult to interpret the risk calculation results, especially for those with a low literacy level.

By using the Django MVC web application framework, together with the backend MySQL database, our CDSS has the flexibilities and the extensibilities of updating the content and modifying the questionnaire. It also makes the system transformable to other applications. For example, by changing the questionnaire contents and the risk score calculation, we can modify and reposition our CDSS for different cancer types, such as breast cancer and stomach cancer.

With the easy-to-follow design of the CRC risk assessment steps, our CDSS embeds scientific CRC risk score calculations into a user-friendly interface. This feature ensures the accessibility of our CDSS to the low literacy population. In our CDSS, a user does not need to have any prior knowledge of CRC risk factors and screening methods for CRC. The system provides all the information on CRC risks and screening in an intuitive way. The innovative risk factor dashboard with customizable stacked bar chart further facilitates the readability and interpretation of the CRC risk level prediction results.

The capability of online appointment scheduling in our CDSS makes it easier to create a link between our CDSS and any EHR in hospitals. After the user fills the risk factors and receives the CRC risk assessments, they can directly make appointments with proper providers with their specific requirements of locations and preferred provider characteristics. The recommendations of appropriate screening methods will be available to the EHR system with original scientific questionnaire data. This feature could assist care providers preparing better before seeing a patient and making more precise care decisions based on patient-specific health conditions.

Given the current system is only a prototype of the CRC CDSS, there are several future directions can be carried out based on the current system.

- For our system, one crucial ongoing task is to perform a systematic evaluation of the CDSS [18], before implementing into a production version. We will work with a working group of patients, providers, health care organizations, and HIT professionals. Multiple-step evaluation processes will be carried out. For instance, we will follow the Software Development Life Cycle for the development and evaluation of the CDSS [19]. A system-wide review of its performance and stabilities will be assessed by IT professionals. On the other hand, the different influential factors and risk calculation algorithms will be validated and evaluated by CRC experts. Also, the interactive website design, the dashboard visualization, and system usabilities will be evaluated with potential users (including patients and providers) for better user experience.
- For healthcare providers, one potential future improvement of the CDSS is to connect the CDSS with different EHR systems. In this way, our CDSS can support effective adoption and achieve health IT interoperability goals. It is also possible to allow each patient to create the patient account so that the system can provide individualized preliminary CRC risk reports based on our interactive dashboard.
- With the development of omics technologies and genomic data analysis, we can integrate the genetic factors or biological factors into our CRC CDSS to expand the assessment function. For instance, based on the gene expression profile, a seven-gene signature has been discovered to predict the overall survival (OS) of CRC patients [20]. We can adopt or modify the survival risk score system to CRC risk score calculation, which could potentially be integrated into our CDSS to improve the accuracy of CRC risk score prediction.

With all these existing features of our CDSS and potential upgrades, we believe our CRC CDSS would be a valuable patient-oriented tool in CRC preventative care field.

## Conclusion

In this study, we have developed a CRC CDSS prototype which gives risk assessment and interactive interpretation of the risk outcomes using innovative data visualizations for personalized CRC screening. The demonstration project is deployed online with Heroku web application deployment platform [21]. The patient-oriented design of our CDSS will help more people to assess their CRC risk and learn more about the significant impact of lifestyle on the development of CRC. Moreover, with the easy-to-follow steps of our CDSS, patients can conveniently build a connection with hospitals and physicians and book their screening test appointments. This feature will make a significant difference in the preventative care of CRC.

## Additional file

Additional file 1: Database structure description in detail. (TXT 2 kb)

## Abbreviations

CDSS: clinical decision support system
CRC: colorectal cancer
EHR: electrical health record
ER: entity relationship
FIT: fecal immunochemical test
